# Reproduction of *Pisidium casertanum* (Poli, 1791) in Arctic lake

**DOI:** 10.1098/rsos.140212

**Published:** 2015-01-28

**Authors:** Yulia Bespalaya, Ivan Bolotov, Olga Aksenova, Alexander Kondakov, Inga Paltser, Mikhail Gofarov

**Affiliations:** 1 Institute of Ecological Problems of the North, The Ural Branch of the Russian Academy of Sciences, Severnaya Dvina Emb. 23, 163000 Arkhangelsk, Russian Federation; 2 Northern (Arctic) Federal University, Severnaya Dvina Emb. 17, 163002 Arkhangelsk, Russian Federation

**Keywords:** adaptive strategy, Arctic, asynchronous brooding, embryonic growth, *Pisidium* species, adaptive coin-flipping

## Abstract

Freshwater invertebrates are able to develop specific ecological adaptations that enable them to successfully inhabit an extreme environment. We investigated the brooding bivalve of *Pisidium casertanum* in Talatinskoe Lake, Vaigach Island, Arctic Russia. Here, quantitative surveys were conducted, with the collection and dissections of 765 molluscs, on the basis of which analyses on the brood sacs length (marsupia) and the number and size of embryos, were performed. In this study, the number of brooded embryos was positively correlated with the parent's shell length. The number of extramarsupial embryos was much lower than the number of intramarsupial embryos. Our research also showed that the brood sac length and embryos within one individual can vary significantly. Thus, we detected that *P. casertanum* has a specific brooding mechanism, accompanied by asynchronous development and embryos release by the parent. We suggest that such a mode could result in the coin-flipping effect that, presumably, increases the population breeding success in the harsh environment of the Arctic lake.

## 2. Introduction

In the Arctic, where the environmental conditions are extreme (i.e. the lake is frozen to the bottom) for the hydrobionts which inhabit freshwater ecosystems, there is only a short summer season, allowing the growth and reproduction of invertebrates [1,2]. Clearly, the species occurring in the Arctic environment have appropriate ecophysiological and life-history trait adaptations to these harsh conditions [3]. A review of the literature shows that the adaptation ability of invertebrates with respect to habitat in the Arctic has been actively investigated recently. For instance, it was revealed that freshwater insects have developed a suite of adaptations, morphological, behavioural, ecological, physiological and biochemical to survive at their physiological temperature minimum [4–6]. Owing to the short summer, many Arctic freshwater invertebrates show prolonged development and live for 2 or more years at high latitudes. By contrast, in temperate regions, the same or closely related species have annual life cycles or more than one generation each year [3,7,8]. In summary, life cycles may become closely adapted to, and synchronized with, the local environmental conditions [3].

The species of the bivalve genus *Pisidium* are frequently dominant in lakes within the Arctic region (Y. V. Bespalaya 2014, unpublished data), they are important ecosystem components and often constitute a large portion of the benthic communities [9]. *Pisidium*species are simultaneous hermaphrodites, synchronously incubating their progeny in brood sacs inside the inner gills and extramarsupially on later stages [9–11]. Some authors presume that evolution of brooding explains the success of sphaeriids in colonizing extreme freshwater habitats [12,13]. Therefore, they are an excellent taxon for studying adaptive reproductive biology [14].

On the other hand, the number of works devoted to the study of the ecology and adaptive mechanisms, which allow freshwater bivalves to successfully inhabit high latitudes, is low. Exceptions are some works dedicated to *Pisidium* species living in Nordic European countries [15,16] and in the Russian European Artic [17,18]. Hence, it was established that in species of the genus*Pisidium* in a single brood sac can be simultaneously found the embryos in different developmental stages [19,20]. This mechanism was termed intramarsupial suppression [19]. It was earlier shown that the number of extramarsupial larvae in *Pisidium* species is much lower than the number of initial embryos, and parents use intramarsupial suppression of embryos to control the number of released juveniles [19]. There is also a possibility that siblings within a brood sac can suppress the development of each other [20,21], which is in accordance with observations indicating that the growth of small embryos in a sac may be retarded and may function as nutrient sources for the larger embryos [22]. Greater mortality of larvae occurred during early, rather than later, stages of larval development [19]. Some authors consider the intramarsupial suppression to be a powerful strategy which is used by the parent to control the number of progeny [11,20].

*Pisidium casertanum* is the most common *Pisidium* species and it is truly cosmopolitan, being distributed worldwide [12,23]. Populations of this mollusc are reported in habitats, ranging from ephemeral ponds to large lakes [23–25] and this species is commonly used as a biological indicator [20,23].

In this study, we aimed to investigate the brooding of *P. casertanum* (Poli, 1791) in Talatinskoe Lake, Vaigach Island, the dominant species of the bivalve community (Y. V. Bespalaya 2014, unpublished data). Based upon the literature, we predicted that the brooding of *P. casertanum* might become closely adapted and synchronized with the local environment dynamic at high latitudes. While investigating this phenomenon, we accounted for the initial embryos in the brood sacs, and made an assessment of the timing and synchronization of the release of juveniles. We also estimated the length and number of embryos for each individual.

## 3. Material and methods

### 3.1 Study area

**Figure 1.**
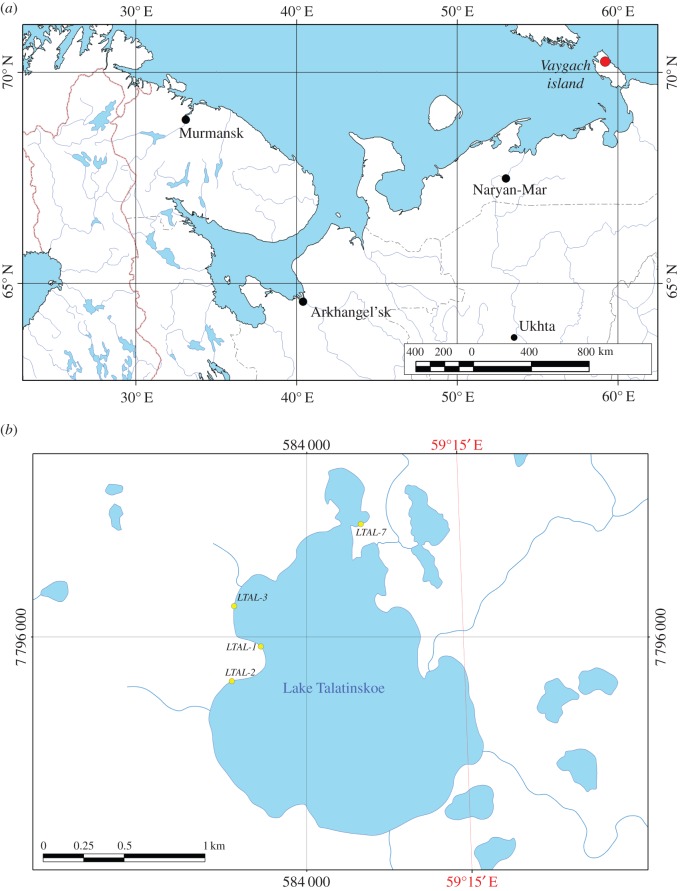
(*a*) Map of the study area. The red circle indicates the location of Talatinskoe Lake on Vaigach Island, Arctic Russia. (*b*) Map of Talatinskoe Lake, Vaigach Island, with the indicated sample locations (yellow circles).

The studies were conducted in the northern part of Vaigach Island (figure 1*a*). The island is located on the border of the Barents and Kara Seas, between South Island of the Novaya Zemlya Archipelago and the Ugorskiy Peninsula [26]. Vaigach Island is separated from the mainland by the Yugorskii Shar Strait and from South Island by the Karskie Vorota Strait, with average altitudes of 50–100 m above sea level. The island is characterized by strong waterlogged territory and a wide distribution of peat bog areas [26,27]. The relief is of coastal plain bordering a shore strip along the periphery of most of the island and upland ridge. According to geological data, Vaigach Island belongs to the thrust structures of the Pai-Khoi and Polar Urals, such as the Novaya Zemlya Archipelago. The Palaeozoic rocks which are overlain by Quaternary deposits are the most widespread geological structures on the island [26,28]. The territory belongs to the Arctic tundra zone [29,30] and is characterized by an Arctic climate [28]. The duration of the ice-free period is *ca* 2.5–3.0 months, from early July to late September [27]. The average air temperature of the warmest month is +5°C in August and the coldest month is −18.5°C in February [28,31].

Mollusc samples were collected from Lake Talatinskoe (70°13^′^ N; 59°14^′^ E) during 3–25 August 2010 which is one of the largest lakes of the island (length 2.2 km, width 1.6 km) belonging to the basin of the Talata-Karskaya River [26]. The lake is shallow, with maximum depth not exceeding 1.5 m, and prevalent depth is *ca* 0.5 m, owing to its thermokarst origin. In winter, the lake freezes to the bottom [32]. The lake has snow-related atmospheric nutrition which it, essentially, enters during intensive snowmelt [26].

### 3.2 Field sampling

Overall, 48 benthic samples were taken at four stations (Ltal-1, Ltal-2, Ltal-3, Ltal-7) (figure 1*b*). For each site, three to six replicates of *P. casertanum* specimens of all size classes were collected using a rectangular hand net (0.28×0.5 m). At three stations, replicates were performed in triplicate every 10 days (3 August, 13 August, 23 August) in order to study the brooding of *P. casertanum* [33]. Samples were washed using a hydrobiological sieve (mesh size 0.56 mm) fixed with 96% ethanol in the field and transported to the laboratory for sorting and identification.

### 3.3 Morphology of shells, brood sacs and embryos estimates for *Pisidium*

**Figure 2.**
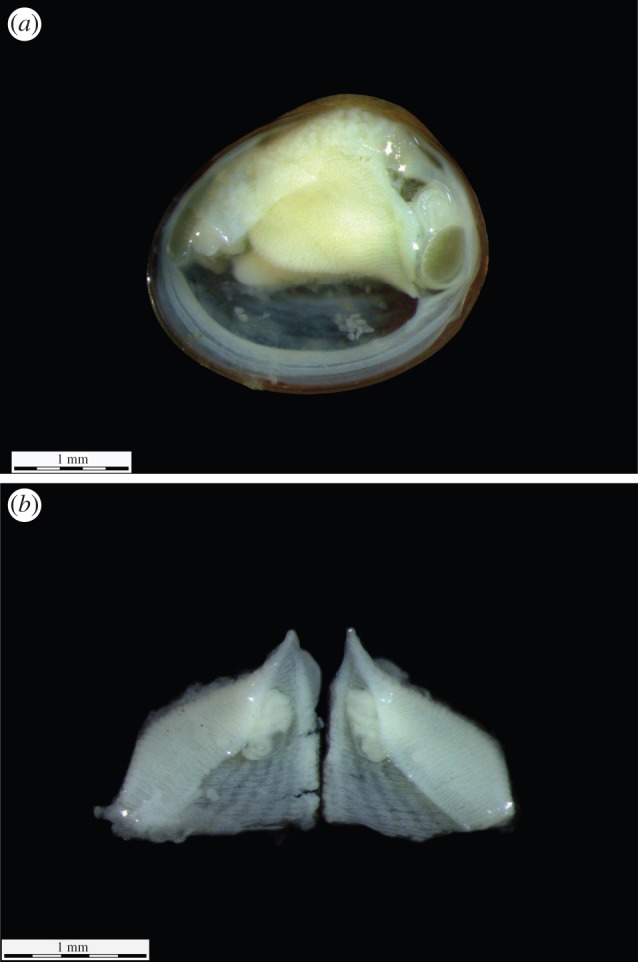
(*a*) The internal morphology dissected individuals of *P. casertanum* (Talatinskoe Lake, Vaigach Island), (*b*) left and right brood sac.

We examined a total of 795 of *P. casertanum* specimens in the laboratory using a stereomicroscope (Leica M165C, Leica Microsystems). All specimens were measured and then dissected in order to record the ratio of gravid animals in each length class and the presence of brood sacs and embryos (figure 2). The estimated stages of sexual maturity were recorded, taking into account the approaches used by other researchers [14,34]. We defined as mature specimens (having embryos), those individuals with shell length of at least 2.2–4.2 mm and the juvenile specimens (lacking the offspring) as non-gravid individuals with shell length of less than 2.2 mm. In total, 139 specimens were gravid, among which 95 individuals had formed two brood sacs. The remaining individuals were not studied because 20 individuals had extramarsupial larvae (i.e. individuals which have broken free from the brood sacs) and 24 individuals had one brood sac at the formation stage.

The left and right marsupial sacs were measured with respect to maximum length and then dissected in order to estimate the number of embryos per sac which was used to denote all classes found within an adult. Deviations were calculated for the sac pair of each specimen (*n*=95) as *D*=SL_max_−SL_min_; where SL_max_ and SL_min_ are maximal and minimal sizes, respectively, of each sac pair. Embryos were removed from marsupial sacs and measured using the greatest dimension by using a microscope with a stage micrometre. All measurements were performed separately for the right and left sacs. In the case where the embryos were at an undeveloped stage (less than or equal to 0.05 mm), their measurements were not performed.

Photographs of the shell, sacs and embryos were produced under a microscope (Leica M165 C) with an attached digital camera (Leica DFC 425, Leica Microsystems). The morphological types of the ontogenetic stages of sphaeriidae were described according to Meier-Brook [19] and Heard [35], with some additions, including the following four classes: class 1: greater than or equal to 0.05 mm, found in brood sacs which are lacking a shell or any definitive shape, usually, a cellular ball; class 2: fetal larvae: 0.2–0.4 mm, clams found in brood sacs which are lacking a shell, but having a definitive shape, such as development of the foot and visceral mass; class 3: prodissoconch larvae: 0.5–0.7 mm, clams found in brood sacs with a shell in various stages of development, but below the minimum birth size; class 4: extramarsupial larvae: greater than 0.8 mm, individuals which have broken free from the brood sacs which are fully developed. There were significant differences between the parameters of the number of embryos by size classes versus size class of parental shell and these were estimated based on the Kruskal–Wallis (multiple comparisons) test.

## 4. Results

### 4.1 Structure of the population and embryonic growth

**Figure 3.**
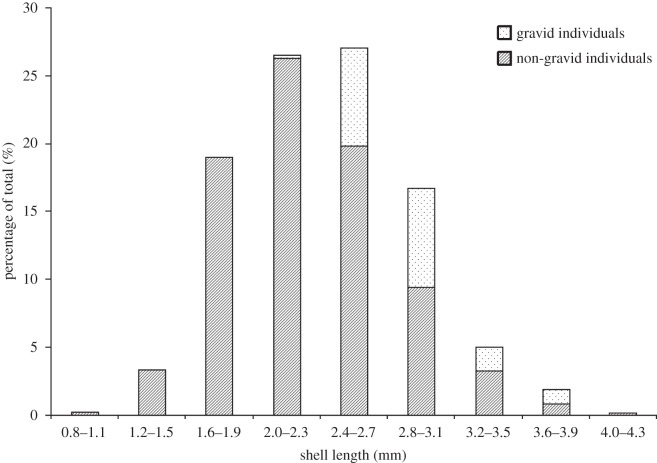
Size frequency structure of the *P. casertanum* sample with proportion of gravid and non-gravid individuals (*n*=795). A total of 139 specimens (17.5% of sample) were gravid.

The size frequency structure of the *P. casertanum* population is presented in figure 3. The maximum shell length of *P. casertanum* in Talatinskoe Lake was 4.3 mm. The average shell length of juveniles at birth was 1.1 mm (0.8–1.6 mm) (*n*=63). The proportion of juvenile (pre-reproductive) to mature bivalves was 49% to 51%, respectively, in our total sample. Among mature bivalves, the percentage of gravid molluscs was 17.5%. Furthermore, the percentage of gravid individuals with shell length 2.2–3.1 mm was 14.7% and with shell length 3.2–3.9 mm was 2.8%.

According to our data, the brood sacs with embryos in the examined population are formed when the shell length of molluscs is at least 2.2 mm. Note that the production of the brood sacs, which depends upon the location (right or left gill), does not occur simultaneously.

**Figure 4.**
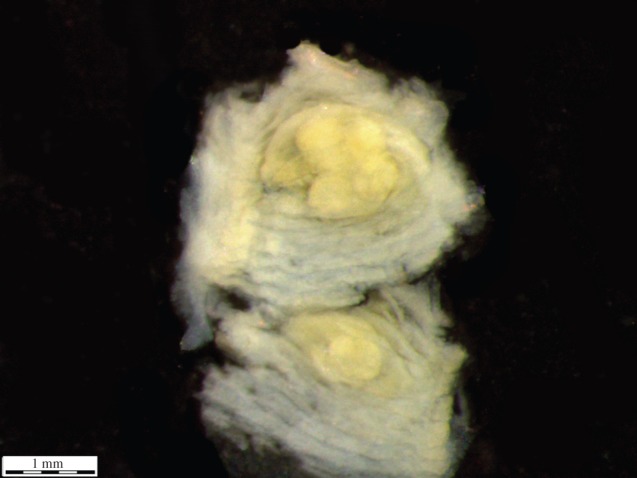
The left and right brood sac dissected individuals of *P. casertanum* (Talatinskoe Lake, Vaigach Island).

**Figure 5.**
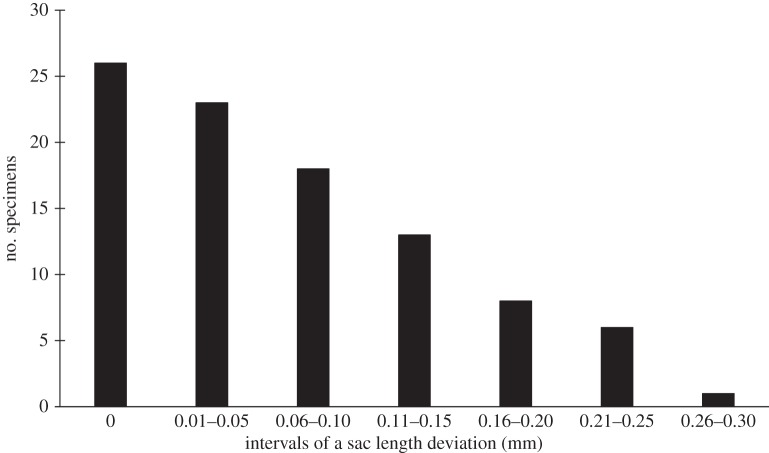
Frequency histogram of sac length deviation of *P. casertanum*. Deviations were calculated for each specimen (*n*=95) as *D* = SL_max_−SL_min_; where SL_max_ and SL_min_ are maximal and minimal length, respectively, of sac pair. A total of 69 specimens (72.6% of sample) have asymmetric development of sacs with mean *D* ± s.d. = 0.104 ± 0.07 mm, min–max = 0.02–0.28 mm.

**Figure 6.**
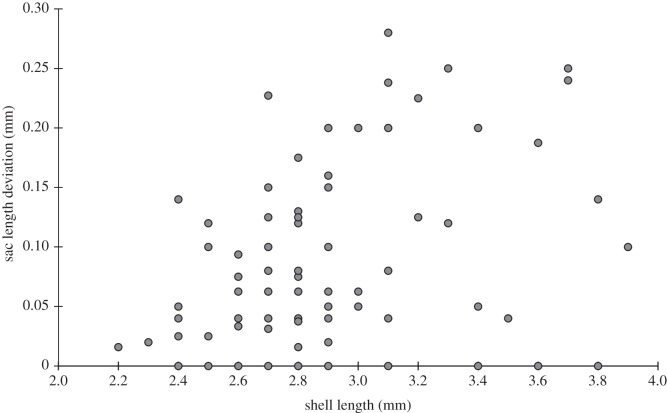
Sac length deviation versus freshwater bivalve shell length of *P. casertanum*. Deviations were calculated for sac pair of each specimen (*n* = 95) as *D* =SL_max_−SL_min_; where SL_max_ and SL_min_ are maximal and minimal length, respectively, of sac pair. Deviation values are not connected with shell length of *P. casertanum* (*R*^2^=13%).

**Figure 7.**
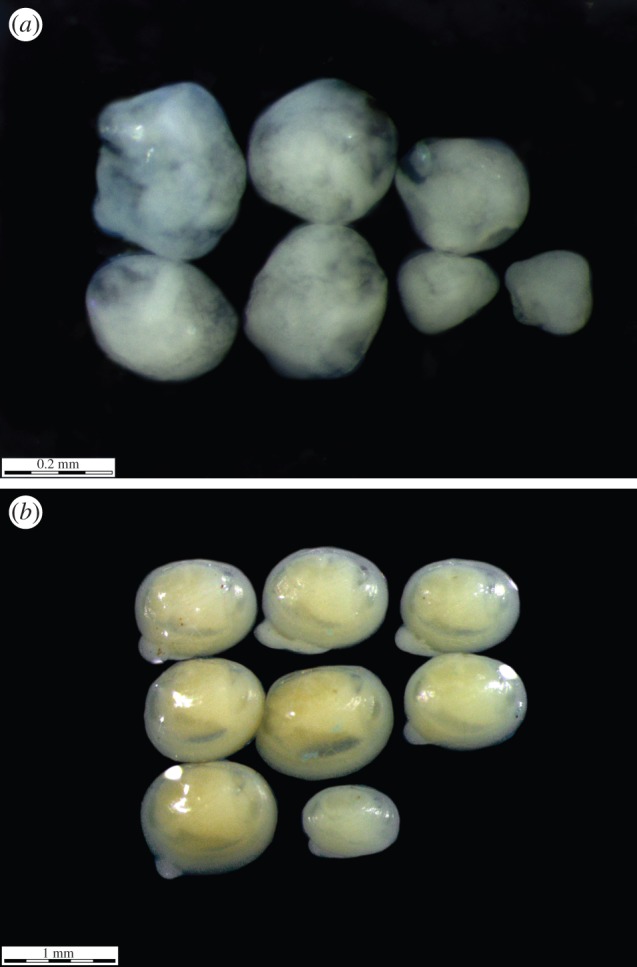
Variations in embryo size within an individual of *P. casertanum*: (*a*) embryos of one to two classes (length 0.05–0.4 mm), (*b*) embryos of three to four classes (length 0.5 to greater than 0.8 mm).

In some cases, the development of the brood sacs was localized at only one of the gills. The frequency histogram of sac length deviation of *P. casertanum* shows that a total of 69 specimens out of 95 studied (72.6% of individuals) have an asymmetric development of sacs with mean *D* ± s.d. = 0.104 ± 0.07 mm, min–max=0.02−0.28 mm (figures 4–6). The mean number of embryos of one to two classes in individuals with shell-length classes of 2.4–3.1 mm was 3.6–6.2 (table 1), and with shell-length classes of 3.2–3.9 mm was 4–12.5. The mean number of embryos of three to four classes in individuals with shell-length classes 2.4–3.1 mm was 1.0–3.1, and with length of shells 3.2–3.9 mm was 3.2–7. The difference between the number of embryos of the 1–4 size classes versus shell-length class are significant (Kruskal–Wallis test: *H* (*χ*^2^)=14.04, *p*<0.003). Variations in embryo size within individuals were also observed (figure 7). It was found that, on several occasions, the embryos of the different sizes have been found in one parental individual.

**Table 1.** Mean number of embryos by size classes versus shell-length class.

	size classes of embryos															
	class 1				class 2				class 3				class 4			
length class of shell (mm)	mean number of embryo	min–max	s.d.	*n*	mean number of embryo	min–max	s.d.	*n*	mean number of embryo	min–max	s.d.	*n*	mean number of embryo	min–max	s.d.	*n*
2.4–2.7	3.9	1–8	2.2	14	5.1	1–10	2.5	16	1	1–3	1	3	2.8	2–4	1.09	5
2.8–3.1	3.6	1–10	2.6	16	6.2	3–11	2.6	17	1	n.a.	n.a.	1	3.1	1–8	2.3	9
3.2–3.5	10.7	6–17	5.7	3	6.8	1–24	8.5	6	4.7	2–7	2.5	3	3.2	2–5	1.6	5
3.6–3.9	4	3–5	1.4	2	12.5	9–16	4.9	2	7	n.a.	n.a.	1	5	n.a.	n.a.	1

## 5. Discussion

### 5.1 Seasonal cycle of reproduction

**Figure 8.**
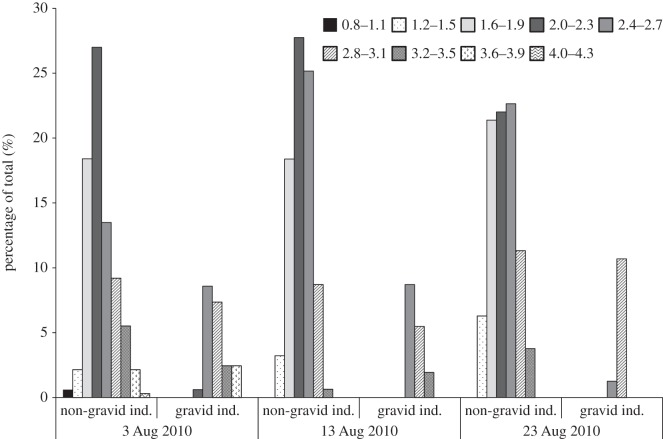
Frequency histogram showing the variation in the length allocation of shells, gravid and non-gravid, of *P. casertanum* during August 2010. *n*=795.

During the study period, the population had a high proportion of juvenile individuals (up to 50%) (figure 8). This suggests that birth occurs between July and August and the breeding season can probably begin in July. Our results are in accordance with observations of the earlier studies [15,21,23,36,37], where *P. casertanum* from populations in Europe and North America presented only a single period of reproduction per year in the spring and summer months. On the other hand, in Denmark, two periods of reproduction for *P. casertanum* were detected in particular years, one in March–April and a second at the end of October [15]. The obtained data on the size structure of the population of *P. casertanum* in Lake Talatinskoe, generally, corresponds to the size parameters of this species in other parts of its distribution range [15,16]. During early August, the population of *P. casertanum* in Lake Talatinskoe is represented by individuals of all size classes (figure 8). Thus, the largest individuals (3.6–4.3 mm) compose a minimum ratio. In mid-August, there was a notable mortality in the oldest classes of *P. casertanum* (figure 8). By the end of August, the number of gravid animals was reduced from 21 to 12%. As can be seen in figures 3 and 8, juvenile and mature individuals have approximately equal proportions during the sampling period.

### 5.2 Reproductive strategies

Sphaeriidae have a highly specialized reproductive system [16], being simultaneously hermaphrodites and viviparous with either synchronous or sequential brooding [9,12,35,38]. *Pisidium* species are synchronous brooders, developing embryos are in a brood sac that is formed by an outgrowth of the ctenidial lamellae [10,20,39]. *Sphaerium* and *Musculium* species are sequential brooders [10,39].

It is known that the Sphaeriidae show much variation in many life-history traits [16,20], such as age at first reproduction, time of egg-laying, time of embryo release, litter size, number of generations per season and others [16]. The reproductive strategies adopted by the Sphaeriidae may differ considerably between species, geographical location and type of environment, depending on ecological factors [20,40]. During conducted comparative analyses of the seasonal dynamics of reproduction of sphaeriid clams from ephemeral and permanent ponds in Ohio (USA) [22,41], it was established that the clams which survive in the harshest environment have a higher number of reproductive strategies. In this study, in the ephemeral ponds there was prolongation of maturation time, which gave a decrease of four to five times in the number of offspring, when compared with the permanent ponds [22]. There is also a noted earlier period of spawning embryos and shortening of duration of release for *Pisidium obtusale* (Lamarck 1818) in ephemeral ponds (i.e. River Volga basin, Russia) which was considered as an adaptation to the early stages of a temporary pond dessiccation process [33,42]. Simultaneously, in the population of *P. casertanum* inhabiting an ephemeral pond (Ontario, Canada) the litter size was larger and the generation time was shorter than that of a conspecific population in a lake [43]. In general, specific behaviour is adopted by sphaeriid bivalves under distinct stressors such as high temperature and desiccation as seen in [42,44–46].

It is well known that the Arctic contains some of the most inhospitable habitat which is colonized by freshwater biota [2]. The environmental conditions are extreme for hydrobionts which inhabit those specific freshwater ecosystems, since there is a short ice-free period [1,3,47]. Consequently, for such conditions the time of brooding is reduced. It is known that temperature is the leading factor in the development of embryos [15,40]. During the short Arctic summer, there are periods of cooling when the temperature can drop to below 0°C [27]. It has been suggested [16,48] that populations living under favourable conditions are likely to be semelparous, while populations living under unfavourable conditions are likely to be iteroparous. According to Pettinelli & Bicchierai [40], the northern populations are located in the middle Palaearctic range of the species, where iteroparous behaviour with only one litter per year seems to be a common feature. Probably, in Talatinskoe Lake, where the ice-free period is only of three months duration and the lake freezes to the bottom, to be semelparous would be irrational, however, this situation requires additional research.

**Figure 9.**
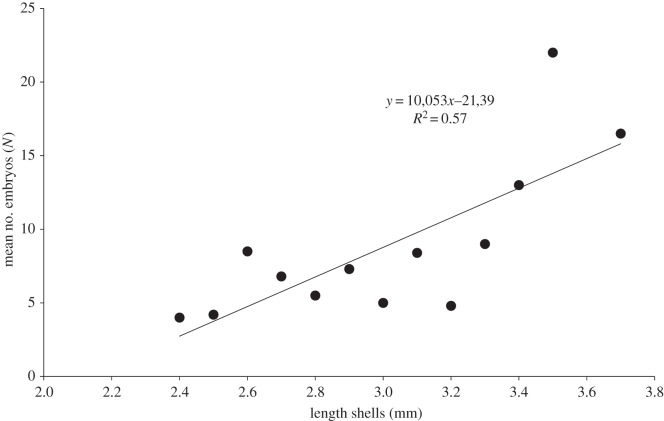
Mean number of embryos versus shell length of *P. casertanum*.

According to our results, the number of embryos is correlated with the parent shell length, i.e. the greater the length of shell of the parent individuals, the higher the number of embryos (table 1 and figure 9), this agrees with previous observations in [14,15,37,49,50]. In [50,51], it is a ‘crucial factor in determining offspring survivorship’ [50], p. 274.

We established that the number of extramarsupial embryos is much lower in *P. casertanum* than the number of initial embryos (table 1). Many researchers, studying brooding of *Pisidium* species, obtained similar results [11,15,19,40]. It has been noticed in [19] that the number of eggs laid is much higher than the number of embryos which attain birth size (i.e. about half of the embryos stop growing at a length of about 0.2 mm and then die) which is a typical feature for sphaeriids. However, the specified size is not consistent with observations by Araujo *et al*. [14] and also by the present data (see the electronic supplementary material, appendix S1; table 1).

Our research also showed that embryo length within one parent individual can vary significantly (figure 7; see the electronic supplementary material, appendix S1). The obtained data agree with the observations of [19,20], where the authors found embryos of various sizes in the *Pisidium* species from the mountain lakes. In fact, work on several species of *Pisidium* described in [19,20] suggest that a chemical component, dissolved in the liquid of the brood sacs, might play a role as a growth inhibitor which is similar to those substances which effect regulation of population density in natural communities. By contrast, studies [33] on the life cycles of *Pisidium* species in ponds of the Volga Basin (Rybinsk Reservoir, Russia) and in the North American Great Lakes [37] found that all of the embryos were always at the same stage of development and only differed slightly in size.

Probably, in unpredictable environments with dramatic fluctuations of temperature, ephemeral ponds, drought, flood and other factors, the body size of larvae and their rates of development will have a strong influence on larval survivorship and these are precisely the characteristics that egg size most profoundly affects [48,52,53]. In addition, it is known that egg size variation is a characteristic that is susceptible to optimization by selection, in response to environmental unpredictability [52,53].

According to the theory [52,54], a female can produce eggs of very different sizes in dependents with environmental factors that increase the reproductive success [48,52–54]. This mechanism was termed a ‘coin-flipping’ strategy, in which an individual is genetically programmed to ‘flip a coin’ before the spring and to choose its egg size, according to the outcome of the toss [54], p. 401. Currently, the reproductive strategy of ‘adaptive coin-flipping’ has been studied on boar [48], plant parts [53], aphids [55], wasps [56] and others.

Our results indicate that release of extramarsupial embryos, presumably, occurs throughout the breeding season from July to September (i.e. before the freeze). Presumably, the laying of embryos in brood sacs occur simultaneously [10]. Then, as the embryos grow, some of them are lagging behind in their development, with respect to the others. Thus, we are likely to witness demonstrations an ‘adaptive coin-flipping’ of reproductive strategy. Therefore, adaptive strategies of arctic freshwater bivalves, in this case are used purposefully with respect to the support of population survival during the breeding period. In these conditions, release of embryos is not simultaneous, which is typical for representatives of the genus *Pisidium* [9–11,22,57], nor is the release occurring after a certain period of time, which would improve the chances of offspring survival.

## 6. Conclusion

The *P. casertanum* population in Talatinskoe Lake probably has iteroparous reproductive tactics with a single period of summer reproduction. Hence, with respect to the freshwater mollusc *P. casertanum* in the Arctic lake of Talatinskoe, there is a specific process of breeding, accompanied by asynchronous development and spawning of embryos. This reproductive strategy aims to improve the breeding success of the population within this extreme environment. This agrees with data from other populations with known stressors, such as in various ephemeral ponds, as well as rivers and lakes, that are exposed to extreme environmental factors. However, it is in contrast to the *Pisidium* species from the environmentally stable freshwater habitats of temperate latitudes which are characterized by synchronous brooding [10]. Clearly, our data fit a ‘coin-flipping’ strategy, where the egg size may be selected according to environmental cues [52].

Futures studies will include the different reproductive tactics of freshwater molluscs which depend upon environmental factors, including the ‘coin-flipping’ strategy.

## Supplementary Material

Number and mean length of embryos by length class versus shell length. In the appendix presents the measured dimensions of mollusk shells and embryos.

## Data accessibility

The datasets supporting this article have been uploaded as part of the electronic supplementary material.
