# Supplementary material for: Reproduction of Pisidium casertanum (Poli, 1791) in Arctic lake
Source: R Soc Open Sci. 2015 Jan 28;2(1):140212. doi: 10.1098/rsos.140212 (PMC4448796; doi:10.1098/rsos.140212)
Supplement: Number and mean length of embryos by length class versus shell length. In the appendix presents the measured dimensions of mollusk shells and embryos. [file rsos140212supp1.docx]

Appendix S1. Number and mean length of embryos by length class versus shell length.

| n/n | Length of shell, mm | Mean length of embryos, mm | Length of embryos, mm (min-max) | SD | Length classes of embryo | | | | Number of embryos |
| --- | --- | --- | --- | --- | --- | --- | --- | --- | --- |
|  |  |  |  |  | class 1 | class 2 | class 3 | class 4 |  |
| 1 | 3.30 | 0.7 | 0.4-0.8 | 0.12 | - | 1 | 7 | - | 8 |
| 2 | 3 | 0.27 | 0.14-0.4 | 0.08 | 2 | 9 | - | - | 11 |
| 3 | 2.6 | 0.21 | 0.16-0.28 | 0.05 | 5 | 5 | - | - | 10 |
| 4 | 2.4 | 0.16 | 0.15-0.18 | 0.01 | 4 |  | - | - | 4 |
| 5 | 2.5 | 0.17 | 0.14-0.24 | 0.05 | 5 | 2 | - | - | 7 |
| 6 | 2.6 | 0.18 | 0.12-0.25 | 0.04 | 5 | 2 | - | - | 7 |
| 7 | 3.5 | 0.16 | 0.06-0.25 | 0.06 | 17 | 5 | - | - | 22 |
| 8 | 3.1 | 1.18 | 1.1-1.25 | 0.06 | - | - | - | 5 | 5 |
| 9 | 2.9 | 0.2 | 0.2-0.24 | 0.01 | - | 10 | - | - | 10 |
| 10 | 2.5 | 0.17 | 0.15-0.18 | 0.01 | 5 |  | - | - | 5 |
| 11 | 2.5 | 1.14 | 1.08-1.2 | 0.08 | - | - | - | 2 | 2 |
| 12 | 3.2 | 1.33 | 1.28-1.38 | 0.07 | - | - | - | 2 | 2 |
| 13 | 2.7 | 0.18 | 0.14-0.2 | 0.03 | 5 | 3 | - | - | 8 |
| 14 | 3.3 | 0.2 | 0.15-0.25 | 0.05 | 6 | 4 | - | - | 10 |
| 15 | 2.5 | 0.36 | 0.25-0.5 | 0.11 | - | 4 | 2 | - | 6 |
| 16 | 3.4 | 0.54 | 0.22-0.78 | 0.27 | - | 3 | 5 | - | 8 |
| 17 | 2.6 | 0.19 | 0.14-0.3 | 0.06 | 8 | 3 | - | - | 11 |
| 18 | 2.4 | 1.1 | 1.1-1.5 | 0.03 |  |  | - | 4 | 4 |
| 19 | 3.1 | 0.29 | 0.18-0.35 | 0.07 | 2 | 8 | - | - | 10 |
| 20 | 2.7 | 0.24 | 0.16-0.3 | 0.05 | 3 | 7 | - | - | 10 |
| 21 | 2.8 | 0.22 | 0.11-0.3 | 0.08 | 2 | 4 | - | - | 6 |
| 22 | 2.8 | 1.56 | n/a | n/a | - | - | - | 2 | 2 |
| 23 | 3.1 | 0.2 | 0.16-0.25 | 0.03 | 6 | 3 | - | - | 9 |
| 24 | 3.2 | 1.58 | 1.56-1.6 | 0.03 | - | - | - | 2 | 2 |
| 25 | 2.9 | 0.2 | 0.16-0.26 | 0.02 | 2 | 11 |  | - | 13 |
| 26 | 2.6 | 0.17 | 0.13-0.25 | 0.05 | 7 | 5 |  | - | 12 |
| 27 | 2.7 | 0.34 | 0.22-0.5 | 0.1 |  | 7 | 3 | - | 10 |
| 28 | 2.7 | 0.24 | 0.16-0.26 | 0.07 | 1 | 5 |  | - | 6 |
| 29 | 3.1 | 0.21 | 0.18-0.22 | 0.02 | 2 | 8 | - | - | 10 |
| 30 | 3.4 | 0.23 | 0.2-0.25 | 0.02 | - | 24 | - | - | 24 |
| 31 | 2.7 | 0.24 | 0.2-0.3 | 0.03 | - | 10 | - | - | 10 |
| 32 | 2.8 | 0.13 | 0.09-0.16 | 0.03 | 4 | - | - | - | 4 |
| 33 | 2.8 | 0.13 | 0.08-0.17 | 0.03 | 7 | - | - | - | 7 |
| 34 | 2.9 | 0.18 | 0.15-0.23 | 0.02 | 7 | 3 |  | - | 10 |
| 35 | 2.9 | 0.18 | 0.15-0.24 | 0.02 | 2 | 8 |  | - | 10 |
| 36 | 2.7 | 0.21 | 0.18-0.24 | 0.02 | 1 | 5 |  | - | 6 |
| 37 | 3 | 0.28 | 0.23-0.48 | 0.09 | - | 6 | 1 | - | 7 |
| 38 | 2.9 | 1.18 | 0.9-1.6 | 0.23 | - | - | - | 8 | 8 |
| 39 | 2.9 | 0.21 | 0.14-0.26 | 0.04 | 1 | 7 | - | - | 8 |
| 40 | 2.6 | 0.25 | 0.22-0.28 | 0.03 | - | 8 | - | - | 8 |
| 41 | 2.8 | 0.25 | 0.16-0.38 | 0.07 | 1 | 6 | - | -- | 7 |
| 42 | 2.7 | 1.18 | 1.16-1.2 | 0.03 | - | - | - | 2 | 2 |
| 43 | 2.9 | 1 | n/a | n/a | - | - | - | 2 | 2 |
| 44 | 2.7 | 0.88 | 0.85-0.95 | 0.05 | - | - | - | 4 | 4 |
| 45 | 2.7 | 0.23 | 0.19-0.32 | 0.07 | 2 | 1 | - | - | 3 |
| 46 | 3.2 | 1 | 0.97-1.1 | 0.05 | - | - | - | 5 | 5 |
| 47 | 2.9 | 1.14 | 1.12-1.16 | 0.02 | - | - | - | 4 | 4 |
| 48 | 3.8 | 1.09 | 0.84-1.19 | 0.14 | - | - | - | 5 | 5 |
| 49 | 3.9 | 0.62 | 0.5-0.72 | 0.07 | - | - | 7 | - | 7 |
| 50 | 3.7 | 0.2 | 0.13-0.25 | 0.03 | 3 | 9 | - | - | 12 |
| 51 | 3.4 | 0.85 | 0.75-0.94 | 0.07 | - | - | 2 | 5 | 7 |
| 52 | 3.7 | 0.22 | 0.11-.32 | 0.05 | 5 | 16 | - | - | 21 |
| 53 | 2.9 | 0.56 | n/a | n/a | - | - |  | 1 | 1 |
| 54 | 2.5 | 0.68 | n/a | n/a | - | - | 1 | - | 1 |
| 55 | 3.1 | 0.23 | 0.16-0.23 | 0.05 | 4 | 7 | - | - | 11 |
| 56 | 2.8 | 0.22 | 0.18-0.26 | 0.04 | 2 | 4 | - | - | 6 |
| 57 | 3 | 1.17 | 1.13-1.19 | 0.04 | - | - | - | 3 | 3 |
| 58 | 2.6 | 0.2 | 0.14-0.24 | 0.04 | 3 | 7 | - | - | 10 |
| 59 | 3.1 | 0.16 | 0.12-0.18 | 0.02 | 10 |  | - | - | 10 |
| 60 | 3.2 | 0.19 | 0.15-0.25 | 0.03 | 9 | 4 | - |  | 13 |
| 61 | 3.2 | 1.1 | 1.1-1.15 | 0.04 | - | - | - | 2 | 2 |
| 62 | 2.8 | 0.2 | 0.17-0.24 | 0.02 | 5 | 3 | - |  | 8 |
| 63 | 3 | 1.2 | 1.1-1.3 | 0.1 | - | - | - | 2 | 2 |
| 64 | 2.7 | 0.2 | 0.18-0.22 | 0.01 | 1 | 8 | - | - | 9 |
| 65 | 3 | 1.25 | n/a | n/a | - |  | - | 1 | 1 |
| 66 | 2.6 | 1.06 | 0.9-1.18 | 0.17 | - |  | - | 2 | 2 |
| 67 | 3.1 | 0.25 | 0.21-0.3 | 0.04 | - | 4 | - | - | 4 |
| 68 | 2.8 | 0.21 | 0.2-0.23 | 0.01 | - | 4 | - | - | 4 |
